# Three dimensional Graphene aerogels as binder-less, freestanding, elastic and high-performance electrodes for lithium-ion batteries

**DOI:** 10.1038/srep27365

**Published:** 2016-06-06

**Authors:** Zhihang Chen, Hua Li, Ran Tian, Huanan Duan, Yiping Guo, Yujie Chen, Jie Zhou, Chunmei Zhang, Roberto DUGNANI, Hezhou Liu

**Affiliations:** 1 State Key Laboratory of Metal Matrix Composites, School of Materials Science and Engineering, Shanghai Jiaotong University, China; 2 Collaborative Innovation Center for Advanced Ship and deep-Sea Exploration, Shanghai Jiao Tong University, China; 3 University of Michigan- Shanghai Jiaotong University Joint Institute, China.

## Abstract

In this work it is shown how porous graphene aerogels fabricated by an eco-friendly and simple technological process, could be used as electrodes in lithium- ion batteries. The proposed graphene framework exhibited excellent performance including high reversible capacities, superior cycling stability and rate capability. A significantly lower temperature (75 °C) than the one currently utilized in battery manufacturing was utilized for self-assembly hence providing potential significant savings to the industrial production. After annealing at 600 °C, the formation of Sn-C-O bonds between the SnO_2_ nanoparticles and the reduced graphene sheets will initiate synergistic effect and improve the electrochemical performance. The XPS patterns revealed the formation of Sn-C-O bonds. Both SEM and TEM imaging of the electrode material showed that the three dimensional network of graphene aerogels and the SnO_2_ particles were distributed homogeneously on graphene sheets. Finally, the electrochemical properties of the samples as active anode materials for lithium-ion batteries were tested and examined by constant current charge–discharge cycling and the finding fully described in this manuscript.

Because of the high specific energy, long cycle life and environmental protection, LIBs have become the main supporting power for all kinds of advanced portable electronic products. With the development of new electrode materials and technology innovations, LIBs which have high charge-discharge rate and specific capacity, have shown great advantages in the electronic field^1^,^2^,^3^,^4^. It has been reported that several kinds of nanostructured, transition-metal oxides^5^ such as SnO_2_ nano-particles can improve the performance of rechargeable LIBs^6^. Single layer, two dimensional graphene has a significantly larger theoretical capacity than graphite and is favorable to lithium ion transport resulting in improving the overall performance of LIBs. But, it is well-known that when graphene is used as lithium-ion batteries electrode materials, the powerful *π*-*π* stacking interactions and Van Der Waals forces make graphene sheets easily aggregate into powders or films which in turn induce higher resistance for Li^+^ ion diffusion and decrease surface area significantly^7^,^8^. Graphene aerogels (GA) display three dimensional porous frameworks, large specific surface area, rapid ion-diffusion characteristics, excellent mechanical strength as well as multidimensional continuous electron-transport pathways and have been fabricated and designed for energy storage and conversion, adsorbents for environmental remediation and catalysis^9^,^10^. Moreover, nano-porous structures could provide additional channels for Li^+^ ions and electrons transmission during the lithiation and delithiation processes^11^,^12^. Three dimensional graphene could offer space for ions expansion and electrolyte storage^8^,^13^,^14^,^15^. In order to take advantage of these features displayed by graphene aerogels, scientists have recently paid increasing attention to the combination of graphene aerogels and LIBs.

Currently, most studies describe graphene hydrogels preparation by hydrothermal method^5^,^16^,^17^, which transfers the mixture solution into a poly (tetrafluoroethylene) (Teflon)-lined autoclave (50 mL) heated at 160 to 200 °C for 5 to 24 hours. Since such preparation method requires relatively high temperature it clearly is not viable for industrial production. Moreover, it has been a recent practice for graphene aerogels to be grinded into powder and mixed with several kinds of conductive agents and binders when using graphene as the cathode material for lithium-ion batteries^18^,^19^. Jun Zhang^20^ fabricated SnO_2_/graphene composites used as anode material by a hydrothermal method with a process heating to 200 °C for 24 h. What is more, the composites were grinded into powder and then mixed with acetylene carbon black (15 wt%) and polyvinylidene fluoride(10 wt%) in N-methyl-2-pyrrolidone (NMP). Lastly, vacuum treatment for 12 h was required to prepare working electrods. Nonetheless, this strategy could not take full advantage of the virtues of GA as clearly the grinding process disrupts the natural GA’s 3D framework. Additionally, conductive agents and binders are indispensable in this type of manufacturing hence increasing the weight of the batteries and complicating the manufacturing process. Thus, such method not only has a lengthy manufacturing process, but also cannot take fully advantage of the aerogel as a three dimensional conductive network.

Herein, we proposed and developed a simple yet novel strategy to fabricate porous SnO_2_-reduced graphene aerogels which have the merits of excellent cycling performance, environmental friendliness, and simple manufacturing process. In the synthesis process for graphene hydrogels, graphene oxide was reduced in a simple mold at low temperature (75 °C). Moreover, Sn-C-O bonds between SnO_2_ nanoparticles and GS were formed after annealing at 600 °C, which will initiate synergistic effect as well as improve the electrochemical performance. Graphene aerogels could then be used as LIBs electrode materials without any further processing or binder addition. Such freestanding and binder-less 3-D graphene with a remarkably high specific surface area and due to the porous structure and an excellent conductivity^21^ was fabricated with a molding technology, which could simplify the preparation for LIBs electrodes as well as avoid adding superfluous material. These auxiliary additives and binder would result in a “dead surface” and a larger resistance^22^,^23^. In this strategy, GA was fully used which would reduce the weight of the batteries and was low-cost. Moreover, these 3D graphene structure could alleviate irreversible restacking and agglomeration caused by the powerful π-πstacking interactions and Van Der Waals forces, as well as promote the transportation of electrolytes within the electrodes^21^,^24^.

## Results

Three dimensional and cylindrical SnO_2_/graphene aerogels which could be used in LIBs without any conductive agent and binder was successfully prepared and characterized in this work. In the proposed manufacturing method, the significantly lower SnO_2_/graphene self-assembly temperature of 75 °C (reduced from typical values of 180 °C or more than 180 °C^25^,^26^) is expected to bring significant savings to industrial production. According to the SEM and TEM imaging, a uniform set of SnO_2_ nanoparticles with diameter in the range of 4~10 nm were firmly anchored on the graphene sheets likely resulting in improved electrochemical properties of the material. Such a three-dimensional porous framework was shown to effectively eliminate SnO_2_ aggregation, provide room for particles expansion and facilitate lithium ions and electrons transport. Also, the synergistic effect between flexible graphene layers and nano-sized SnO_2_ improve the capacity and cyclic stability. The described superior properties are believed to result mainly form the high specific surface area and the excellent conductivity of the electrode material.

## Discussion

Homogenous SnO_2_ dispersion was prepared through a hydrothermal method^27^,^28^,^29^. SnO_2_-GA three dimensional porous aerogels were fabricated by a hypothermal self-assembly strategy which we believe to be more suitable for industrial production.

SnO_2_ particles with positive surface charge have a tendency to attract negatively charged oxygen-containing functional groups on graphene oxide^11^. Thus, SnO_2_ nanoparticles with a diameter of 4~10 nm would adhere to the surface of graphene sheets (GS) tightly as a result of electrostatic attraction^30^,^31^. Moreover, because of the strong electrostatic attraction, SnO_2_ particles will remain anchored on the surface of graphene sheets after sonication and stirring. The dark brown color of the SnO_2_-GO dispersion suggested that the SnO_2_ nanoparticles were adhering directly onto the graphene sheets. Subsequently, L-ascorbic acid was used as reducing agent to transform *in situ* Graphene Oxide (GO) into reduced GO (rGO)^8^. As a result of the decrease of oxygen-containing groups on the surface of GS and driven by π-π stacking interactions, GO anchored with SnO_2_ particles was reduced to GS and self-assembled into a 3D hydrogel. Such reaction would result in the increase and enlargement of delocalized π-bond’s conjugative effect^32^. After these reactions, various oxygen containing functional groups were found to be left on the surface of rGO nano-sheets. Since the SnO_2_ nanoparticles had polar surfaces, they interacted with functional groups via hydrogen bonding^6^,^27^,^30^. Such hydrogen bonding could transform into oxygen bridges between SnO_2_ and rGO by forming Sn-O-C bonds after annealing at 600 °C. Moreover, such bridges would stabilize the SnO_2_ nanoparticles on the graphene surface, moreover, facilitate the electron, transfer and improve the electrode stability^4^,^33^ and initiate synergistic effect.

**Figure 1:**
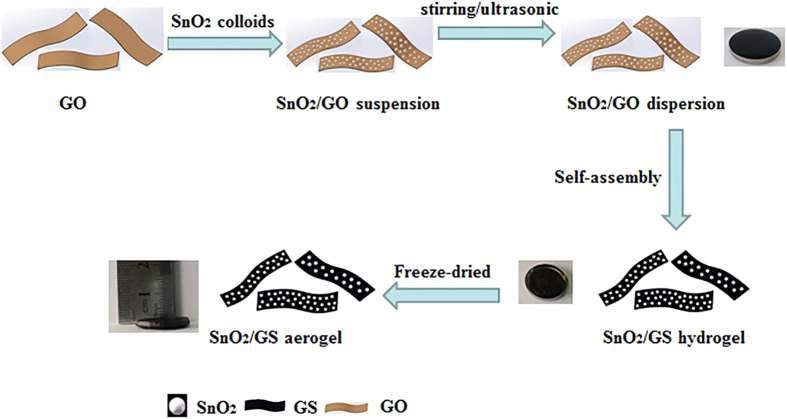
Schematic synthesis procedure of the preparation of the SnO_2_-GA and SnO_2_-rGA.

It is known that graphene aerogels have excellent conductivity and mechanical strength which suggests that graphene aerogels could be directly used as electrodes without any additional additives^13^,^34^. The synthesis procedure of SnO_2_/Graphene hydrogels is schematically shown in Fig. 1 by hypothermal hybridization. After freeze-drying, graphene aerogels where obtained in shape and sizes comparable the ones obtained by GHs.

**Figure 2:**
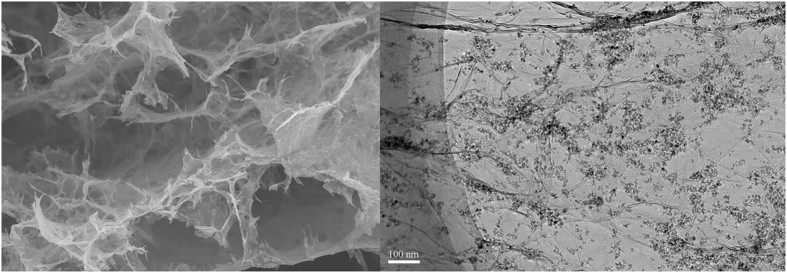
(**A**) SEM image of SnO_2_-GO composite at low magnification with a flake. (**B**) TEM image of SnO_2_-GO composite. (**C**) TEM image of SnO_2_-GO composite after one charge/discharge cycle.

The morphologies of SnO_2_-GA were characterized both by scanning electron microscopy (SEM) and transmission electron microscopy (TEM). Figure 2(A) shows an SEM image of the laminar graphene aerogels, which displays an interconnected and porous morphology. The size of the pores ranged from 10 μm to 50 μm with the pore walls consisting of thin layers of graphene sheets formed during the self-assembly of graphene. The TEM images (Fig. 2(B)) show that the size of SnO_2_ nanoparticles were approximately in the range of 4 nm to 10 nm, moreover, the nanoparticles appeared to anchor on the GS firmly and homogeneously.

Figure S(1) shows the Raman spectra of SnO_2_-rGA and SnO_2_-GA. For SnO_2_-rGA; the two prominent peaks at ~1339 cm^−1^ and ~1576.5 cm^−1^ corresponded to D and G bands, respectively. For SnO_2_-GA, the G-band was around 1338 cm^−1^ and the D bands was around 1577 cm^−1^. The intensity ration of the D to G peak (I_D_/I_G_) increased from 1.199 to 1.283, which could be explained by the size decrease of the sp^2^ domain and confirmed the reduction of graphene oxide after heat treatment^35^,^36^.

**Figure 3:**
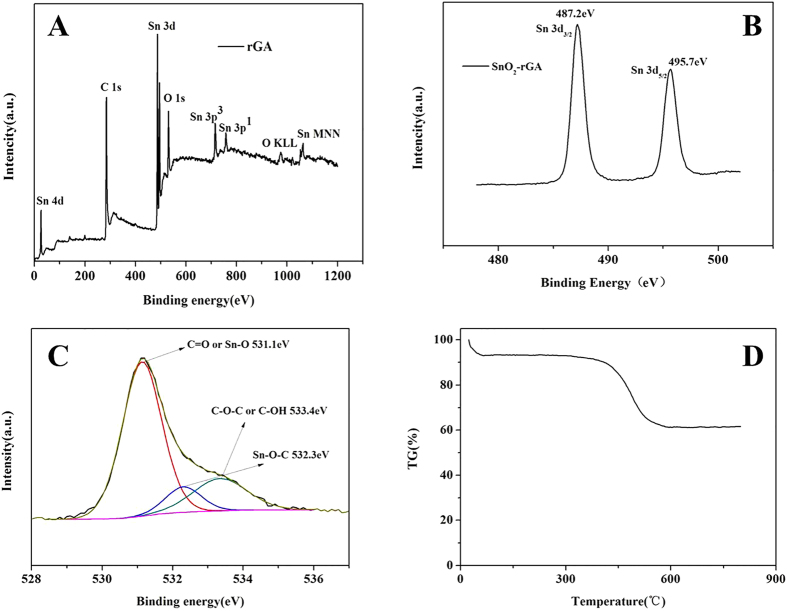
(**A**) High resolution XPS spectra of SnO_2_-rGA; (**B**) Sn 3d XPS spectrum of SnO_2_-rGA; (**C**) O1s XPS spectrum of SnO_2_-rGA; (**D**) The TGA between 20 °C and 800 °C.

X-ray photoelectron spectroscopy (XPS) was carried out to confirm the surface chemical state and the chemical elements in the framework after annealing. According to the high-resolution scan spectrum (Fig. 3(A)), it was found that the sample only contained C, O and Sn. The peaks of C1s, O1s and Sn3d were at 284.6, 530.6, and 486.6 eV^30^,^37^. Figure 3(B) revealed the XPS spectrum of Sn3d. There were two peaks located at 487.2 and 495.6 eV which corresponded to Sn3d_5/2_and Sn3d_3/2_. The peak at 487.2 eV of Sn3d_5/2_ belonged to Sn^4+^ in SnO_2_^7^. This peak confirmed the presence of SnO_2_ on the surface of graphene layers. The peak of O1s was shown in Fig. 3(C). There were three predominant peaks located at 531.1, 532.2 and 533.4 eV, which corresponded to Sn-O and/or C = O bonds, Sn-C-O bonds, and C-OH and/or C-O-C groups (hydroxyl and/or epoxy)^38^, respectively. The Sn-C-O bonds formed after annealing treatment revealed the widespread connection between graphene layers and the SnO_2_ nanoparticles, which could initiate synergistic effect and improve the electrochemical properties^38^,^39^. Before annealing at 600 °C, the connection between SnO_2_ nanoparticles and GS was physisorption. Without any chemical bonds, the interaction between SnO_2_ nanoparticles and GS was Van der Waals force and hydrogen bond, the SnO_2_ nanoparticles may separate form GS during Li^+^ insertion and extraction, resulting in the attenuation of capacity during charge/discharge cycling. After annealing at 600 °C, the hydrogen bonds transform into oxygen bridges and form Sn-C-O bonds, which would strongly anchor the SnO_2_ nanoparticles and initiate synergistic effect. The synergistic effect between GS and SnO_2_ nanoparticles was larger than the contribution of all constitutive components^40^. Moreover, such bridges facilitate the transport and the reversible lithiation and delithiation, leading to a superior cycling stability and rate capability^41^.

The amount of SnO_2_ anchored on the graphene sheets’ surface in the nano-composites was tested by thermogravimetric analysis (TGA) from 25 °C to 800 °C with a heating rate of 5 °C min^−1^ (Fig. 3(D)). A severe weight loss was noted between 400 °C and 550 °C due to the decomposition of the graphene layers. The weight ratio of GA was calculated to be 66.02%.

The specific resistance and the square resistance of SnO_2_-GA was measured to be 650 mΩ cm^−1^ and 7000 mΩ cm^−1^ through a four probes method, suggesting the GA could transport electrons quickly as a result of the three dimensional electrically conducting framework. The excellent measured conductivity is expected to result in a superior electrochemical performance of the electrode.

**Figure 4:**
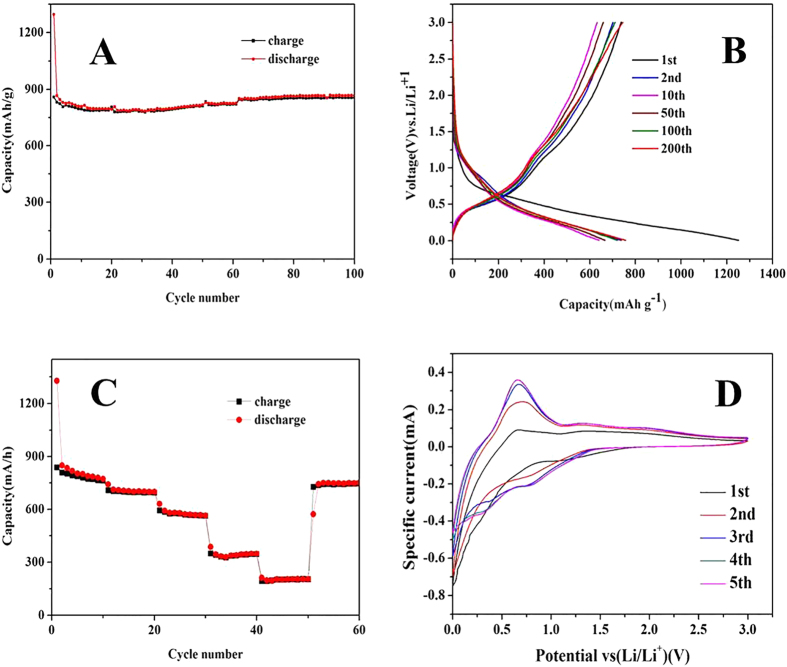
(**A**) Cycling test for the graphene sheet at the current density of 100 mAh g^−1^;(**B**) Discharge/charge profiles (1^st^, 2^nd^, 10^th^, 50^th^, 100^th^, 200^th^ cycle) of the as-prepared graphene sheet at the current density of 200 mAh g^−1^ as a LIB electrode. (**C**) Cycle performance of the graphene sheet as a LIB anode at different current densities. (**D**) CV curves of the graphene sheet as a LIB electrode.

To evaluate the electrochemical properties of SnO_2_-GA systematically, galvanostatic discharge-charge (lithium insertion-lithium extraction) measurements with current density of 200 mA g^−1^ with voltage between 0.01 and 3 V, were carried out. It was noteworthy that the specific capacities were calculated on the basis of the weight of the active nanocomposites materials. Figure 4(A) shows the long cycle performance of SnO_2_-GA using charge-discharge repetition at a current density of 100 mA g^−1^, the typical charge-discharge profiles of SnO_2_-GA in the 1^st^, 2^nd^, 10^th^, 50^th^, 100^th^ and 200^th^ cycles are shown in Fig. 4(B). In the first cycle, SnO_2_-GA displays a discharging capacity of 1295.2 mAh g^−1^ and a reversible lithium de-intercalation charging capacity of 857.9 mAh g^−1^ with coulombic efficiency of 66.2%. In the second cycle, the reversible discharge capacity of SnO_2_-GA was found to be 866.6 mAh g^−1^. Subsequently the value decreases to 783.5 mAh g^−1^ in the 31^th^ cycle and then increased to 867.3 mAh g^−1^ in the 99^th^ cycle. Moreover, the coulombic efficiency was found to be higher than 96.5% after the 3^rd^ cycle. Because the graphene structure was porous and elastic, when graphene nanostructure was compressed and used as electrodes, the porous structure prevented substantial shrinkage. Furthermore, the structure subsequently inflated during the charge/discharge cycles^8^,^29^,^42^. The contact was improved along with the inflation, which could result in the increase of charge/discharge capacity. The rate characteristic of the SnO_2_-GA at different current densities of 100 mA g^−1^, 200 mA g^−1^, 500 mA g^−1^,1500 mA g^−1^, 3000 mA g^−1^ are illustrated in Fig. 4(C). There was almost no decrease of reversible capacity at a low density of 100 mA g^−1^. Furthermore, the capacity not only did not decrease, but even increased at higher current density of 200 mA g^−1^, 500 mA g^−1^, 1500 mA g^−1^ as well as 3000 mA g^−1^. When the current density returned to 100 mA g^−1^, the reversible capacity remained at 750 mAh g^−1^, indicating the nanocomposite displayed an excellent electro-chemical performance. One possible explanation for the superior capability and stable cycling performance of the material was that graphene wafer had a large contact surface area with the electrolyte. Moreover, the lithium ions could be adsorbed to the surface of graphene layers physically during the charging and discharging cycles because of the extensive surface area. The synergistic effect^34^,^43^,^44^ between SnO_2_ nanoparticles and graphene layers as well as the decomposition of electrolyte could contribute to the increase in capacity^45^,^46^. Another reason for the superior performance of the electrode was the presence of SnO_2_ nanoparticles, and the electrical conductivity, and mechanical flexibility of the graphene structure. The uniform distribution of SnO_2_ nanoparticles played a vital role in improving the electrochemical properties of LIBs. Since the graphene wafer prepared was porous and elastic, it could accommodate the volume expansion of SnO_2_ during charge/discharge cycles. In addition, the porous and interconnected graphene nanostructure could provide an effective conduction path for electron transfer and accommodate the mechanical strain caused by the Li^+^ insertion/extraction^41^,^47^.

Figure 4(D) shows the cycle voltammetry curves (CVs) for the first 5 cycles of the SnO_2_-GA between 0 and 3 V vs. Li^+^/Li at a sweep rate of 0.5 mV s^−1^. In the first cycle, the electrode was unstable because no binder was used in the batteries and the contact did not reach an ideal state. After the first cycle, the curves were found to be more consistent. It could be seen from the curves that there were four reduction peaks at 1.21 V, 0.82 V, 0.45 V, ~0.2 V, respectively. Along with the formation of Li_2_O, the transition from SnO_2_ to Sn (eq 1, eq 2) contributed to the pronounced cathodic peaks of 1.21 V and 0.82 V. The peak at 0.45 V could be attributed to the decomposition of the electrolyte and the formation of solid-electrolyte interphase (SEI) on the surface of SnO_2_ and GS^2^,^17^,^48^. The Li-Sn alloying (eq 3) and the lithium-ion insertion on the GS surface (eq 4) caused a reduction peak at around 0.1 V. There were two oxidation peaks at 0.63 V and 1.32 V which were related to the de-alloying of Li_x_Sn (eq 3) and the generation of SnO_2_^3^,^6^,^49^. It is worth noting that the generally considered irreversible reactions (eq1, eq2) became partially reversible reactions because of the ability of the ultra-small size of SnO_2_ to decrease the activation energy^2^,^3^,^50^. After the second cycle, the nearly overlapping CV curves reflected a superior reversibility of lithium insertion and extraction reactions.


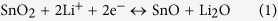



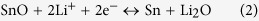



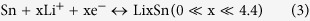



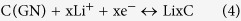


The porous nature of SnO_2_/graphene aerogel composite was demonstrated by Brunauer–Emmett-Teller (BET) measurements as shown in Figure S(2). It could be seen in the figure that the N_2_ adsorption-desorption isotherms exhibited a typical II hysteresis loop as a relative pressure between 0.45 to 0.96, characteristic of pores with different pore sizes. The nitrogen physisorption measurements traced the change of pore structure and the specific area was calculated to be 168 m^2^ g^−1^ while the pore diameter was distributed mainly at approximately 3 nm. These results demonstrated that it is feasible to obtain three dimensional graphene frameworks with solvothermal assembly approach. Moreover, the high porosity provided more surface area for lithium-ions insertion and extraction reactions as well as more room for SnO_2_ expansion.

**Figure 5:**
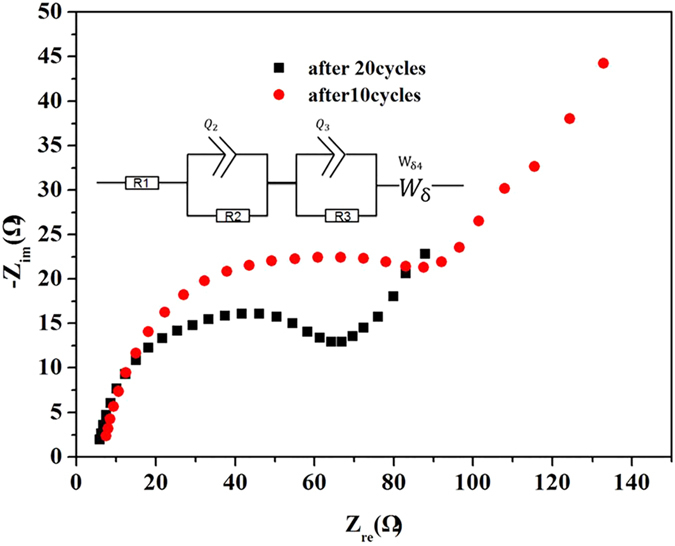
The enlarged high frequency region of the Nyquist plots of SnO_2_-rGO.

Electrochemical impedance spectroscopy (EIS) measurements were utilized to study the electrode’s superior electrochemical performance and the kinetics properties^46^. Figure 5 shows the Nyquist plots of the SnO_2_-rGA after 10 cycles and SnO_2_-rGA after 200 cycles as well as the equivalent circuit model. It was found that R_2_ and R_3_ were due to the solid electrolyte interface impedance and to the charge transfer resistance, respectively. Moreover, the liner part of the plot is likely due to the lithium diffusion in the 3D porous network^46^,^51^. The semicircle diameter of the SnO_2_-rGA-20 was smaller than that of SnO_2_-rGA-10, implying that the prepared electrodes were stable during charge/discharge cycles^52^. Improved charge transfer and inherent impedance characteristics leading to rate capability enhancement are expected to result from the high electrical conductivity^48^.

**Figure 6:**
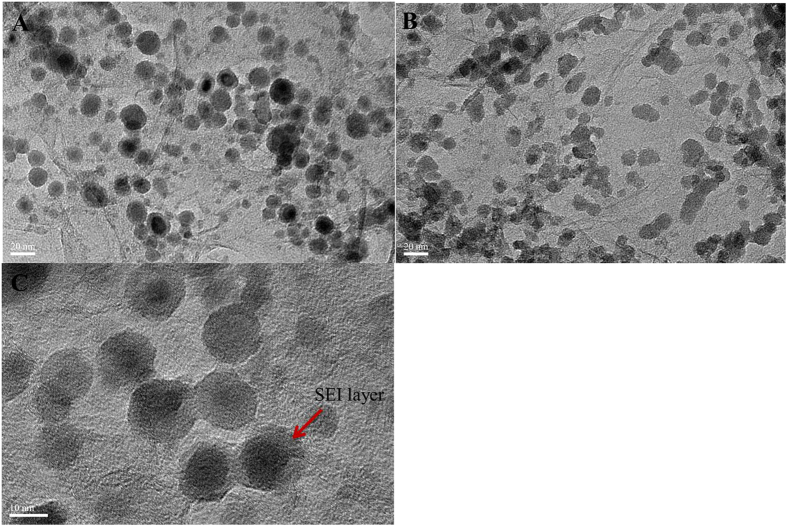
(**A**) TEM image of lithiated SnO_2_-GA composite; (**B**) TEM image of delithiated SnO_2_-GA composite; (**C**) TEM image of lithiated SnO_2_-GA composite.

The TEM images of lithiated and delithiated SnO_2_/GA are shown in Fig. 6. Based on the images, it is clear that a SEI layer formed after discharge caused by the decomposition of the electrolyte. The formation of SEI leads to irreversible capacity loss during the initial discharge/charge cycle due to the consumption of Li ions and electrons. Moreover, the SEI layer existed in the delithiated SnO_2_/GA and eliminated the volume expansion of SnO_2_ nanoparticles, which will lead to an excellent stability, resulting in excellent performance during discharge/charge cycling. Although a SEI layer (Fig. 6(C)) formed on the surface of the graphene layers, it is clear from the images that the SnO_2_ nanoparticles did not aggregate and remained dispersed. The strong interaction between SnO_2_ nanoparticles and graphene layers together with the three dimensional graphene could effectively eliminate the particles aggregation during cycles. All these features observed and described are expected to lead to a superior cycling performance and stability^52^,^53^. Comparing Fig. 6(A,B), we concluded that the volume expansion of SnO_2_ nanoparticles was eliminated resulted from the homogeneous distribution of SnO_2_ nanoparticles on graphene sheets^54^. The robust structure of 3D porous graphene could provide electron transport kinetics for Li ions and buffer the large volume expansion during the long term alloying-dealloying process, prevent the aggregation of SnO_2_ nanoparticles and strengthen the contact between the active material and the electrolyte, which would lead to the enhanced cycle performance and rate capacity^49^,^55^,^56^.

## Methods

### Synthesis of graphene oxide dispersion

Graphene oxide (GO) dispersion was prepared by a modified Hummers method. Briefly, 3 g of graphite powder, 1.5 g of NaNO_3_, and 69 ml of H_2_SO_4_ were mixed in a beaker. 9 g KMnO_4_ were added into the beaker and stirred. An ice-bath was used to keep the temperature of the mixture to 0 °C. After stirring for 30 minutes, the temperature was raised to 35 °C, while stirring for additional 30 minutes. Subsequently, 138 ml of cold (15 °C) de-ionized water was added slowly. Then, 213 ml of warm (80 °C) water and 15 ml of H_2_O_2_ were added. The mixture was separated by centrifugation and washed by HCl solution (HCl/H_2_O = 1/10) three times. At last, the mixture was dissolved in de-ionized water and dispersed homogeneous by ultrasonic agitation for several hours.

### Preparation of SnO_2_ nanocrystal

2.325 g of SnCl_4_·5 H_2_O was dissolved in 200 ml de-ionized water and then hydrothermally treated at 160 °C for 16 hours. The obtained white precipitate was washed by centrifugation three times and re-dispersed in 200 ml ethanol to form a suspension.

### Synthesis of SnO_2_/Graphene aerogels

**Figure 7:**
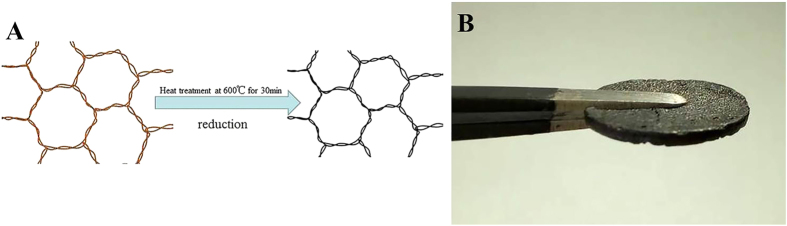
(**A**) Schematic representation of the structure of the GA during annealing treatment; (**B**) Photograph of 3D graphene sheet.

2.4 ml of SnO_2_ dispersion was added drop-wise into 6 ml graphene oxide (2 mg ml^−1^) so that the mass ratio of graphene oxide and SnO_2_ was 1:1. In order to achieve homogeneous dispersion, additional stirring and ultrasonic agitation were carried out for more than 24 consecutive hours. Afterwards, 120 mg of ascorbic acid powder was added into the mixture and stirred for an additional 30 minutes. The dispersion was added into a mold and put into a draught drying cabinet at 75 °C for 4 hours to obtain 3D graphene hydrogels. Subsequently, the monolith was released from the mold and put in de-ionized water for 24 hours, and freeze-dried into graphene aerogel (Fig. 1). Finally, the aerogels was heated at 550 °C for 30 minutes to improve the degree of reduction (Fig. 7(A)).

### Electrode Preparation

The graphene aerogels were heated at 650 °C for 30 minutes under nitrogen atmosphere. After being heated, the 3-D graphene sheets (Fig. 7(B)) was used as electrodes without additives or and post-processing.

### Material Characterization

The morphology of samples was characterized by transmission electron microscope (JEM-2100F, JEOL, Tokyo, Japan). Field-emission scanning electron microscope (FE-SEM) analysis was performed on JSM-6700F at an acceleration voltage of 10.0 kV. X-ray photoelectron spectroscopic (XPS) measurements were performed on a Kratos AXIS Ultra DLD spectrometer with a monochromatic AlKa X-ray source. Thermal gravimetic analysis (TGA) was conducted in oxygen atmosphere at a heating rate of 5 °C min^−1^ from 25 °C to 800 °C. X-ray photoelectron spectroscopy (XPS) analysis was conducted using a Kratos AXIS Ultra DLD spectrometer with a monochromatic AlKa X-ray source. Conductivity was measured by a four-point probe method in the van der Pauw configuration with an Accent HL5500 system. N_2_ adsorption/desorption isotherms were determined by an Auto sorb IQ instrument. The Brunauer-Emmett-Teller (BET) method was carried out to calculate the surface areas.

## Additional Information

**How to cite this article**: Chen, Z. *et al*. Three dimensional Graphene aerogels as binder-less, freestanding, elastic and high-performance electrodes for lithium-ion batteries. *Sci. Rep*. **6**, 27365; doi: 10.1038/srep27365 (2016).

## Supplementary Material

Supplementary Information
