# Supplementary material for: Three dimensional Graphene aerogels as binder-less, freestanding, elastic and high-performance electrodes for lithium-ion batteries
Source: Sci Rep. 2016 Jun 6;6:27365. doi: 10.1038/srep27365 (PMC4893605; doi:10.1038/srep27365)
Supplement: Supplementary Information [file srep27365-s1.pdf]

Supplementary information

Three dimensional Graphene aerogels as binder-less, freestanding, elastic and high-performance electrodes for lithium-ion batteries

Zhihang Chen†[1], Hua Li†[*1, *2], Ran Tian[1], Huanan Duan[1], Yiping Guo[1], Yujie Chen[1], Jie Zhou[1], Chunmei Zhang[1], Roberto DUGNANI[3], Hezhou Liu[*1, *2]

1 State Key Laboratory of Metal Matrix Composites, School of Materials Science and Engineering, Shanghai Jiaotong University

2 Collaborative Innovation Center for Advanced Ship and deep-Sea Exploration, Shanghai Jiao Tong University

3 University of Michigan- Shanghai Jiaotong University Joint Institute

Figure S(1) Raman spectra of SnO_2-GO and SnO_2-rGO

Figure S(2) Nitrogen adsorption–desorption isotherms

Figure S(1)

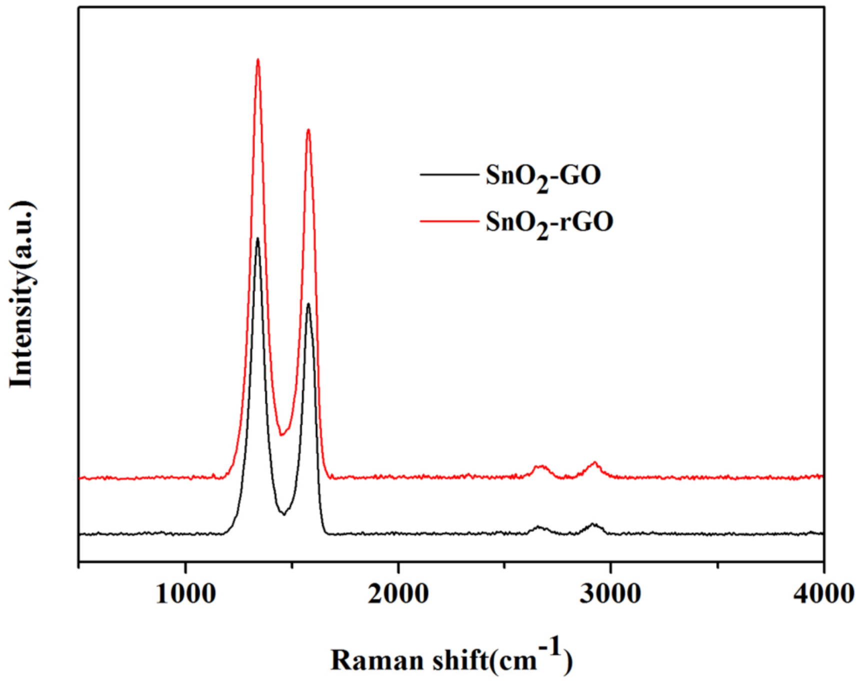

SnO2-GO
SnO2-rGO
Intensity(a.u.)
1000
2000
3000
4000
Raman shift(cm-1)

Figure S(2)

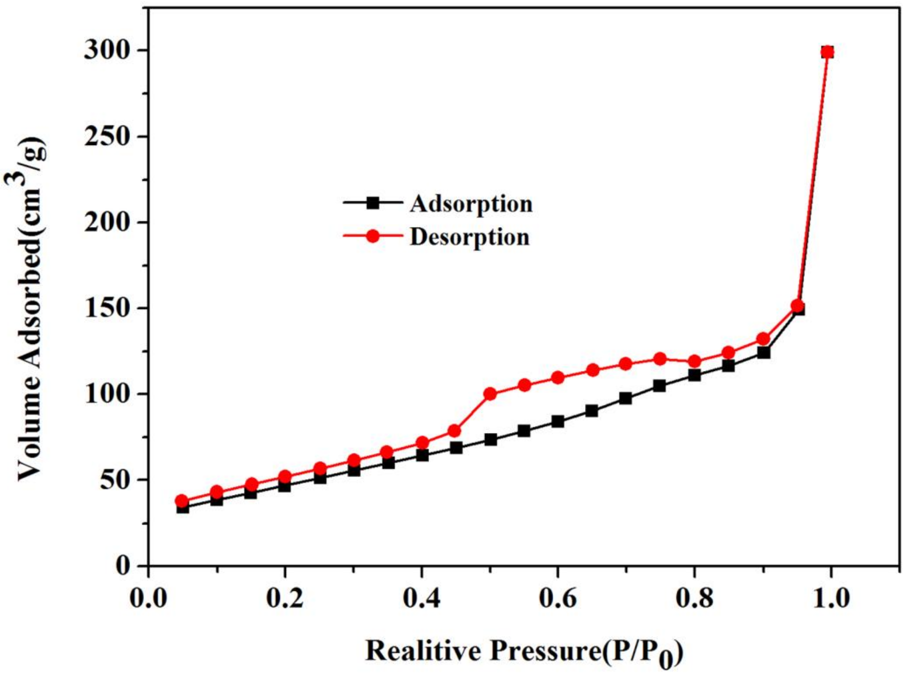

300
250
200
150
100
50
0
0.0
0.2
0.4
0.6
0.8
1.0
Adsorption
Desorption
Volume Adsorbed(cm3/g)
Realitive Pressure(P/P0)
